# Gene Expression Signatures of Peripheral Blood Mononuclear Cells during the Early Post-Transplant Period in Patients Developing Cardiac Allograft Vasculopathy

**DOI:** 10.1155/2010/719696

**Published:** 2010-12-28

**Authors:** Khurram Shahzad, Martin Cadeiras, Sarfaraz Memon, Barry Zeeberg, Tod Klingler, Anshu Sinha, Esteban G. Tabak, Sreevalsa Unniachan, Mario C. Deng

**Affiliations:** ^1^Division of Cardiology, Department of Medicine, College of Physicians and Surgeons, Columbia University, New York, NY 10032, USA; ^2^Division of Cardiology, Department of Medicine, University of Alabama at Birmingham, Birmingham, AL 35294, USA; ^3^Genomics and Bioinformatics Group, Laboratory of Molecular Pharmacology, National Cancer Institute, National Institutes of Health, Bethesda, MD 20892, USA; ^4^XDx Inc., Brisbane, CA 94005, USA; ^5^Courant Institute of Mathematical Science, New York University, New York, NY 10012, USA; ^6^Cardiac Transplantation Research, Center for Advanced Cardiac Care, Columbia University College of Physicians & Surgeons, New York Presbyterian Hospital, PH Room 1291, 622 W 168th Street, New York, NY 10032, USA

## Abstract

*Background*. Cardiac allograft vasculopathy (CAV) is a major cause of graft loss and death after heart transplantation. Currently, no diagnostic methods are available during the early post-transplant period to accurately identify patients at risk of CAV. We hypothesized that PBMC gene expression profiles (GEP) can identify patients at risk of CAV. *Methods*. We retrospectively analyzed a limited set of whole-genome PBMC microarrays from 10 post-transplant patients who did (*n* = 3) or did not (*n* = 7) develop advanced grade CAV during their long-term follow-up. We used significance analysis of microarrays to identify differentially expressed genes and High-Throughput GoMiner to assess gene ontology (GO) categories. We corroborated our findings by retrospective analysis of PBMC real-time PCR data from 33 patients. *Results*. Over 300 genes were differentially expressed (FDR < 5%), and 18 GO-categories including “macrophage activation”, “Interleukin-6 pathway”, “NF-KappaB cascade”, and “response to virus” were enriched by these genes (FDR < 5%). Out of 8 transcripts available for RT-PCR analysis, we confirmed 6 transcripts (75.0%) including FPRL1, S100A9, CXCL10, PRO1073, and MMP9 (*P* < .05). *Conclusion*. Our pilot data suggest that GEP of PBMC may become a valuable tool in the evaluation of patients at risk of CAV. Larger prospectively designed studies are needed to corroborate our hypothesis.

## 1. Introduction

Cardiac allograft vasculopathy (CAV) is a major cause of graft loss and death after heart transplantation (HTx). Identification of surrogate makers for late cardiac allograft survival has been of major interest to improve long-term outcomes of HTx [1]. After HTx, alloantigens (including molecules from donor endothelium) are presented by antigen-presenting cells to the recipient's T-cells, often generating a differentiated inflammatory response. That response includes T-cells, B-cells, and a coordinated pattern of cytokine release. Cells of innate immunity (monocyte-derived macrophages) are also involved [1]. Non-antigen-specific perioperative events including microvascular insults may play pivotal roles related to subsequent development of CAV, probably related to ischemia reperfusion injury, advanced donor age, hyperlipidemia, depletion of arteriolar tissue plasminogen activator factor and systemic inflammation [1–8]. The inflammatory response culminates in migration of mononuclear cells through the coronary vascular endothelium and phenotypic switching of medial smooth muscle cells mediated by generation of growth-promoting cytokines. Those processes contribute to chronic damage of the coronary arteries of the transplanted heart. The result is the development of a diffuse, obliterative form of vasculopathy characterized by production of a “neointima” rich in vascular smooth muscle cells and extracellular matrix [9, 10]. After the first year post-transplantation, 30% to 50% of patients have some evidence of CAV and after 5 years CAV is one of the leading causes of death with <50% 1-year survival rate in those with extended disease [11, 12]. 

Limited interventions have been shown to prevent, delay, or reverse CAV. While no definite well-validated surrogate marker for late cardiac allograft outcome is available, early detection of CAV represents the *key* strategy as an effective surrogate [1]. Early identification of CAV became possible with the introduction of intravascular ultrasound (IVUS) [13], but the technique is invasive, it is usually not initiated until at least one year post-transplantation, is expensive, and requires the use of nephrotoxic contrast agents. Noninvasive tests, including stress perfusion, dobutamine echocardiography, ultrafast tomography, and MRI have not proven to be sufficiently sensitive or specific to detect early stages of the disease [14, 15]. Therefore, there are clear needs to explore and develop new options for the early evaluation of patients at risk of CAV.

Recently, gene expression profiles of peripheral blood mononuclear cells (PBMC) were used to identify patients with [16, 17] or without moderate and severe acute cellular cardiac allograft rejection [18] and patients at risk of antibody-mediated rejection [19]. 

Since the peripheral recirculation of recipient leukocytes after allo-endothelial-cell contact in the allograft may carry information about immune activation conducive to chronic rejection development, we hypothesized that gene expression profiles of PBMC obtained early after HTx carry molecular signatures that correlate with the future development of CAV.

## 2. Materials and Methods

### 2.1. Patients, Samples, and Microarrays

We analyzed a limited set of 41,000 gene expression profiles (whole-genome Microarray, Agilent Technologies, Wilmington, DE) obtained from patients included in a large multicenter study (Cardiac Allograft Rejection Gene Expression Observational [CARGO] study) that used microarrays to identify PBMC gene signatures of acute cellular cardiac allograft rejection [18]. Columbia University Medical Center (CUMC) contributed 121 patients. Out of the Columbia University cohort, independent whole genome PBMC samples from 10 patients were available. The study protocol was approved by the Institutional Review Board (IRB) of CUMC. PBMC were isolated from eight mL of venous blood using density gradient centrifugation (CPT, Becton-Dickinson, Franklin Lakes, NJ). Samples were frozen in lysis buffer (RLT, Qiagen, Valencia, CA) within 2 h of phlebotomy. Total RNA was isolated from each sample (RNeasy, Qiagen, Valencia, CA). Whole genome gene expression profiling was performed on two-color Whole Human Genome 60-mer Oligo Microarrays (Agilent Technologies, Santa Clara, CA), which contain 41,000+ unique transcripts. After the hybridization, data were extracted and Lowess normalized expression files were generously provided by the CARGO study sponsor (XDx Inc., Brisbane, CA). Filtering was done against background and only those probes with more than 1.5-fold change were retained. Probes mapping to the same gene transcript were not averaged.

### 2.2. Cardiac Allograft Vasculopathy

Patients were eligible for the study if they were evaluated with angiography during their post-transplant course. CAV was defined as any evidence of disease in the angiography as evaluated by one of three expert interventional cardiologists who specialized in heart transplantation care at the Center for Interventional Vascular Therapy at Columbia University. Angiograms were classified as normal (no evidence of vasculopathy), mild (any grade of angiographic luminal stenosis less than 50%), moderate (any angiographic luminal stenosis greater than 50% in a main vessel or two secondary branches), and severe (more than 50% in the left main or two main vessels). Patients with moderate or severe CAV were considered “advanced”.

### 2.3. Statistical Analysis

Quantitative and qualitative clinical variables were compared by the Mann Whitney *U*-test, Chi-Square test, or Fischer-exact when appropriate using SPSS 11.5.1 (SPSS Inc., 2002). A *P*-value <.05 was regarded as significant. Gene expression of samples obtained from patients with advanced CAV was compared against those with normal or mild disease using independent, unpaired *t*-test, and Benjamini-Hochberg correction for multiple comparisons to estimate a false discovery rate (FDR) as implemented in significance analysis of microarrays (SAM) [20] after 1.5-fold change filtering. Genes were retained for further analyses if FDR < 5%. Molecular function and biological processes associated with individual significant genes were determined using PANTHER (Protein ANalysis THrough Evolutionary Relationships) Classification System (http://www.pantherdb.org), a unique resource that classifies genes by their functions, using published scientific experimental evidence and evolutionary relationships to predict function even in the absence of direct experimental evidence. Quantitative, real-time PCR cycle thresholds (CT) for each group were compared with the Mann Whitney *U*-test. *P*-value <.05 was considered significant.

### 2.4. Hierarchical Clustering

Gene expression data were correlated and visualized in clustered heat maps [21] using the Genesis web interface ((http://carmaweb.genome.tugraz.at/ genesis/index2.html), Institute for Genomics and Bioinformatics, Graz University of Technology, Graz, Austria). Top 100 genes and microarray samples as well as differentially enriched GO categories and genes were clustered on the basis of co-occurrence of the differentially expressed genes using the Pearson metric and average linkage.

### 2.5. Gene Ontology Analysis

We used High-Throughput GoMiner (HTGM) [22] (http://discover.nci.nih.gov/) to analyze the list of interesting genes in the context of gene ontology (GO) categories [23]. HTGM analyzes data from all microarrays in a study, provides diagnostics for data interpretation and visualization tools in terms of specialized clustered “heat maps” (also called clustered image maps, CIMs) [21]. Normally, the input to HTGM consists of a total-genes file (representing the entire Microarray or a randomly generated whole genome seed) and a changed-genes file (representing the genes with altered expression) relevant to the study purpose. The output generated by HTGM includes a summary of the results, a matrix whose rows are categories and whose columns are names of changed gene for hierarchical clustering of experiments and categories, and a statistical summary for each category including one-sided Fisher exact *P*-value and an FDR. Hierarchical clustering of enriched categories and changed genes allows determining which categories achieved statistical significance by virtue of containing essentially the same set of changed genes.

## 3. Results

### 3.1. Patient Demographics

The baseline characteristics of the study population, including mean age, gender, race, diagnosis, mean cold ischemia time, induction strategy, antirejection prophylaxis, median follow-up time at the time that the microarrays were obtained, median follow-up time till the diagnosis of CAV, simvastatin use, and median number of rejection episodes (i.e., 1R/1B, 2R, and 2R/3A) requiring augmented immunosuppression are depicted in Tables 1(a) and 1(b) for the overall population and by study subgroups and sample method. All baseline clinical characteristics were comparable across groups in both microarray and PCR studies. Although the difference in timing of blood sample post-HTx between advanced CAV and normal/mild CAV groups in the microarray study population approached the defined level of significance (*P* = .06) but this difference was not significant in the PCR study population.

### 3.2. Microarray Analysis

Out of 10 patients who were studied with microarrays, 3 (30%) had advanced CAV while 7 (70%) had angiograms consistent with either absence of disease (*n* = 3, 30%) or mild disease (*n* = 4, 40%). Comparison of study samples for each study group is summarized in Table 1(a). One sample that had 2R (moderate) cellular cardiac allograft rejection was retained given the limited small Microarray dataset.

### 3.3. RT-PCR Analysis

There were 64 Quantitative, 253 transcript, Real-Time PCR samples available from our Center, obtained from 43 patients during the CARGO study [18]. Nine samples from 9 patients associated with ISHLT grade 3A/2R rejection and 1 sample without rejection grading information were excluded. Out of the remaining samples, 3 samples had no angiographic information, 1 sample had aneurismatic disease diagnosed at year 1 without further information in subsequent years, and 18 samples from 13 patients were repeated. For repeated samples, we choose the one closest to the median time from the transplant surgery to the date that all samples were obtained. Therefore, we retained 33 samples obtained from 33 patients for this analysis. Twenty-eight PCR samples (85%) belonged to patients from the normal (*n* = 20) or mild disease (*n* = 8) group, and 5 samples (15%) belonged to patients with advanced CAV.

### 3.4. Gene Expression Profiles

**Figure 1:**
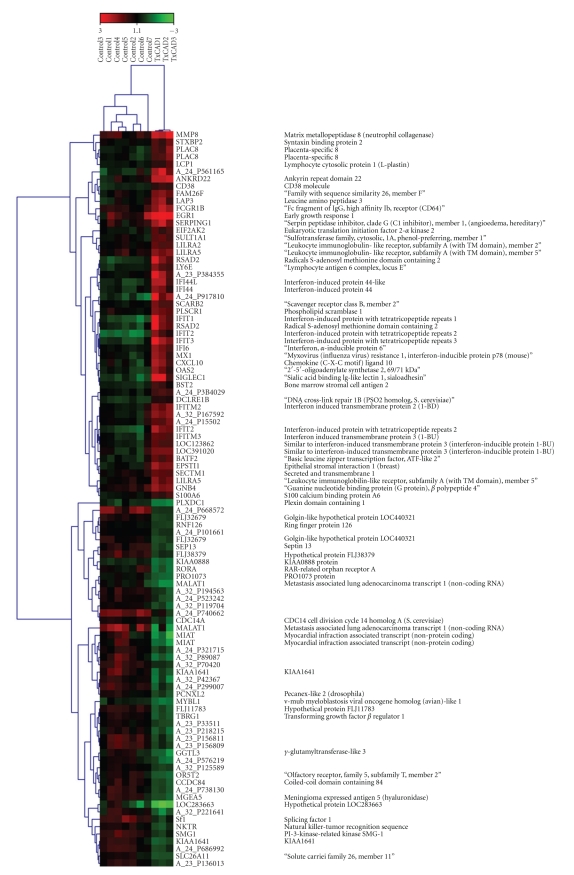
Clustered heat map of top 100 up- and down-regulated genes in patients with advanced transplant coronary artery disease and controls. Gene symbol and gene name are provided when available. Platform unique identifier is provided otherwise.

Between-group comparisons identified 316 transcripts from 291 unique genes differentially expressed with FDR ≤ 5%: 182 transcripts (170 genes) were up-regulated and 134 transcripts (123 genes) were down-regulated. Top up- and down-regulated genes and their functions are depicted in (Table 2) and a clustered heat map of top 100 up- and down-regulated genes is shown in (Figure 1). A complete list of genes with their respective fold changes and false discovery rates is provided as supplementary material available online at doi:10.1155/2010/719696.

### 3.5. Gene Ontology Analysis

**Figure 2:**
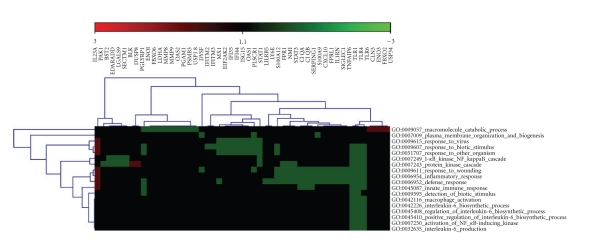
Clustered heat map of enriched GO categories by differentially expressed genes. Instances of cross-talk are shown that links GO categories enriched by similar genes. The green areas show GO categories enriched by the down-regulated genes while small red areas in the corners show GO categories enriched by up-regulated genes.

HTGM detected 18 changed GO categories (FDR < 5%) enriched with differentially expressed genes (Table 3). Processes related to enriched GO categories included among others: macrophage activation, response to wounding, response to virus, response to biotic stimulus, interleukin-6 biosynthetic process, I-kappaB kinase NF-kappaB cascade, innate immune response, inflammatory response, regulation of interleukin-6 biosynthetic process, defense response, positive regulation of interleukin-6 biosynthetic process, activation of NF-kappaB-inducing kinase, and interleukin-6 production. Evaluation of the clustered image map indicates potential cross-talk among GO categories enriched by advanced CAV genes. Instances of cross-talk are of particular importance because they tie together categories that, based on prior knowledge, might be thought of as unrelated. A clustered heat map of differentially expressed genes and enriched gene ontology categories and cross-talk is shown in (Figure 2).

### 3.6. RT-PCR Validation

We compared the list of 291 candidate genes that we identified by SAM based on 5% FDR for this exploratory study with the 252 candidate gene list of the available RT-PCR generated in the Discovery Phase of the CARGO study [18]. We found 8 transcripts (7 different genes) overlapping. The validation rate based on Mann Whitney *U*-test comparison between absence of, or mild CAV (*n* = 28) and advanced CAV (*n* = 5) was 75% (2 not validated out of 8). Validated genes included FPRL1 (*P* = .005), S100A9 (*P* = .005), CXCL10 transcript 1 (*P* = .009), and CXCL10 transcript 2 (*P* = .019), PRO1073 (*P* = .045), MMP9 (*P* = .045). Nonvalidated genes included ENO1 (0.083) and FAS (*P* = .104). SAM-based differentially expressed genes and the corresponding RT-PCR gene *P*-values showed good concordance (Table 4).

## 4. Discussion

A gene signature is the group of genes in a type of cell whose combined expression pattern is a unique characteristic of a disease under investigation. These disease specific gene signatures can be used to select patients at a specific state of a disease with an accuracy that facilitates the diagnosis of the disease and selection of patients for different treatment options.

In this pilot study, patients with advanced CAV show peripheral blood gene expression profiles that differ already in the early period after transplantation from those of patients who will not develop advanced CAV. The findings are consistent with previous suggestions that donor- and recipient-related factors in the perioperative period may play major roles in the immune response and development of a sustained chronic response to immune injury, leading to proliferation of the endovascular matrix, with consequent obstruction of the blood flow and impairment of allograft function [2, 4, 9, 10].

In previous studies, gene expression profiles of PBMC have been shown to correlate with presence or absence of *concurrent* acute cellular rejection [16–18, 24], antibody mediated rejection [19], and in response to mechanical circulatory support device implantation [25, 26]. Based on these gene expression profiles, the genomic classifier developed to rule out acute cellular cardiac allograft rejection has also been shown to correlate with different organ function related parameters of rejection [27, 28], and longitudinal changes in clinical profiles [29, 30]. In current study, we hypothesized that gene signatures early after HTx correlate with *future* development of CAV. To the best of our knowledge, this is the first report of using a high throughput genomic screening approach to identify patients at risk of developing advanced CAV on the basis of leukocyte samples obtained during the early period after transplantation. This observation has potentially important implications. 

Recently CAV has been reported as one of the major causes of new-onset graft dysfunction and associated outcomes [31]. Early detection of CAV was proposed as the *key* strategy, to identify surrogate markers for late cardiac allograft outcomes [1]. Currently, the most accurate diagnostic test to define the phenotype of CAV is intravascular ultrasound [9]. Evaluation of the neointimal proliferation rate between months 3 and 12 has been shown to predict survival and major adverse cardiac events at long-term follow-up [11] but intravascular ultrasound is invasive, expensive and complication prone. Therefore, early profiling of an immune response that differentiates a “high CAV-risk phenotype” from a “low CAV-risk phenotype” may become valuable to early identify these patients, model timely therapeutic interventions and monitor response.

This concept in HTx is not new. Earlier observations in animal models of rejection suggested that “unstable tolerance induction” is associated with the persistent immune activation that mediates destruction of graft parenchymal cells, and that the evaluation of biological processes involved might be useful in identifying different types of rejection and the likelihood of progression to chronic forms [32]. 

In our study, we found differentially expressed genes that significantly enriched GO categories including the innate immunity, macrophage activation, interleukin-6, activation of the NF-KappaB cascade and response to virus. The finding that the innate immune response plays an important role is interesting because it has been already described in the literature. Differentially expressed genes, among others in this category, included Toll-like receptors 1, 4 and 6, and Interleukin-23 alpha. Several authors proposed an important role of innate immunity in the pathogenesis of CAV [10, 33–35]. For example, it has been shown that in patients with allograft endothelial dysfunction (an early clinical indicator of transplant vasculopathy), mRNA transcript level and surface expression of TLR-4 on circulating monocyte is significantly higher than controls [34]. According to our results the categories related to these mechanisms were enriched by the down-regulated genes. The interaction between the up- and down-regulated genes collectively determines the functioning of different biological processes as assessed by high through-put microarray analysis. Our observations show some interesting expression patterns of these mechanisms during the *early* period post-HTx which needs further understanding. Therapy with Simvastatin that inhibits allograft inflammatory activity and attenuates endothelial coronary dysfunction shows reduced trans-cardiac IL-6 and TNF-alpha gradients [33] and polymorphisms within the promoter region of the IL-6 gene has been proposed as risk markers for CAV [36]. In addition, the transcription factor NFKB becomes up-regulated in response to CD40/ CD40 ligand mediating apoptosis of endothelial cells. Inhibition of this pathway has been shown to be protective against the development of CAV [37]. The GO analysis shows that the STAT1 gene involved in the NFKB pathway is also part of the response to virus showing a “cross-talk” between both categories and suggesting that mechanisms of immune damage related to viruses (i.e., CMV) [9, 10] may be related to the NFKB pathway.

The findings presented in this paper should encourage the development of strategies to advance evaluation and management of heart transplant recipients in a preventative, preemptive and personalized way, but the conclusion from this pilot study should be interpreted within the important limitations imposed by the small sample size, the use of angiography which is an imperfect standard for detection of CAV, and the known variable reproducibility of gene expression studies [38]. Corroboration of genes was based on retrospective PCR data. Only a small number of genes were available that were present in the Microarray-based candidate gene list. 

*In conclusion,* our data show that peripheral blood leukocyte genes, specifically innate immune response genes, are differentially expressed during the early time after HTx in patients who develop advanced CAV. Larger prospectively designed studies are needed to corroborate our findings.

##  Disclosure

All authors report no conflict of interests in relationship to the data presented here.

## Supplementary Material

Supplementary Appendix 1: This supplementary material includes the complete list of differentially expressed genes including the probe ID, Gene Symbol and Gene Name along with the information on degree of fold change, q-value, and direction of regulation in advanced CAV as compared to the normal or mild disease.
Supplementary Appendix 2: This supplementary material includes the complete list of the GO categories enriched by the significantly differentially expressed genes, including category names with the number of total and changes genes in individual category. It also includes the one-sided Fisher exact p-value and the false discovery rate as calculated by the HTGM algorithm.

## Figures and Tables

**Table:** (a)

	Microarray study (*N* = 10)			PCR study (*N* = 33)		
Variable	Normal or mild (*N* = 7)	Advanced CAV (*N* = 3)	*P*-value	Normal or mild (*N* = 28)	Advanced CAV (*N* = 5)	*P*-value
Time after HTx (days)	232 (81–298)	42 (41–96)	.067	81 (7–171)	55 (41–167)	.643
Follow-up time (years)	5.3 (4.8–5.8)	5.6 (5.4–5.7)	.138	5.5 (4.6–6.2)	5.7 (5.1–5.8)	.315
Male gender recipient	6 (85.7%)	3 (100%)	1	21 (75.0%)	4 (80%)	1
Male gender donor	6 (85.7%)	2 (66.7%)	1	20 (71.4%)	3 (60%)	.627
Caucasian recipient	5 (71.4%)	3 (100%)	.585	21 (63.6%)	5 (100%)	.903
Caucasian donor	7 (100%)	1 (33%)	.67	17 (60.7%)	2 (40%)	.685
Recipient age	55.6 ± 8.2	48.5 ± 13.5	.383	52.6 ± 105	61.4 ± 8	.173
Donor age	31.7 ± 11.7	44.1 ± 7.9	.183	32.8 ± 13.0	41.5 ± 13.7	.226
ICM recipient	5 (71.4%)	1 (33.3%)	.11	14 (50%)	4 (80%)	.267
LVAD	3 (42.9%)	2 (66.7%)	1	7 (21.2%)	2 (40%)	.597
Recipient CMV (+)	2 (28.6%)	0 (0.00%)	.53	11 (33.3%)	2 (40%)	.347
Donor CMV (+)	3 (42.9%)	2 (40.0%)	.7	14 (50%)	4 (80%)	.445
Ischemic time (Min)	188.7 ± 65.1	138 ± 36.6	.229	183.9 ± 49.2	156 ± 7	.208
Daclizumab induction	7 (100%)	3 (100%)	—	24 (85.7%)	4 (80%)	
Prednisone	7 (100%)	3 (100%)	—	28 (100%)	5 (100%)	—
Prednisone dose	8.71 ± 7.8	13.3 ± 2.9	.296	16.3 ± 10.2	13.0 ± 4.5	.359
Maintenance Regimen			1			.295
Cyclosporine	6 (85.7%)	3 (100%)		24 (85.7%)	4 (80%)	
Tacrolimus	1 (14.3%)	0 (0.0%)		3 (10.7%)	0 (0%)	
Sirolimus	0 (0.0%)	0 (0.0%)		1 (3.6%)	1 (20%)	
Cyclosporine levels (ng/ml)	437.0 ± 489	225.3 ± 26	.655	312.5 ± 226	229.25 ± 22	.212
Tacrolimus levels (ng/ml)	8.5 ± 0.2	—	—	11.7 ± 2.5	—	—
Sirolimus levels (ng/ml)	—	—	—	6	5.6	—
Mycophenolate	6 (85.7%)	3 (100%)	1	24 (85.7%)	5 (100%)	1
Azathioprine	0 (0.0%)	0 (0.0%)	—	0 (0.0%)	0 (0.0%)	—
Simvastatin	1 (14%)	1 (33%)	1.00	2 (7%)	0 (0%)	1.00
Cellular rejection (ISHLT)			.161			.78
0R	4 (57.1%)	2 (66.7%)		24 (85.7%)	4 (80%)	
1R	3 (42.9%)	0 (0.0%)		4 (14.3%)	1 (20%)	
2R	0 (0.0%)	1 (33.3%)		0 (0%)	0 (0%)	
New episodes of rejection						
1R/1B	0 (0–1)	0 (0–0)	.513	0 (0–4)	0 (0–3)	.688
2R	1 (0–1)	1 (0–1)	.789	0 (0–2)	0 (0–1)	.375
2R/3A	0 (0–1)	0 (0–0)	.513	0 (0–2)	0 (0–3)	.898

HTx: heart transplant; LVAD: left ventricular assist device; RT-PCR: real-time polymerase chain reaction.

**Table:** (b)

	Normal or mild			Advanced CAV			Microarray versus PCR (*N* = 43)		
Variable	Microarray (*N* = 7)	RT-PCR (*N* = 28)	*P*-value	Microarray (*N* = 3)	RT-PCR (*N* = 5)	*P*-value	Microarray (*N* = 10)	RT-PCR (*N* = 33)	*P*-value
Time after HTx (days)	232 (81–298)	81 (7–171)	.002	42 (41–96)	55 (41–167)	.571	154.5 ± 99.5	76.4 ± 47.5	.037
Follow-up time (years)	5.3 (4.8–5.8)	5.5 (4.6–6.2)	.433	5.6 (5.4–5.7)	5.7 (5.1–5.8)	.451	5.3 ± 0.3	5.4 ± 0.4	.583
Male gender recipient	6 (85.7%)	21 (75%)	1	3 (100%)	4 (80%)	1	9 (90%)	25 (75.8%)	.659
Male gender donor	6 (85.7%)	20 (21.4%)	.648	2 (66.7%)	3 (60%)	1	8 (80%)	23 (69.7%)	.698
Caucasian recipient	5 (71.4%)	21 (75%)	.781	3 (100%)	5 (100%)		8 (80%)	26 (78.8%)	.823
Caucasian donor	7 (100%)	17 (60.7)	.26	1 (33.3%)	2 (40%)	.641	8 (80%)	19 (57.6%)	.433
Recipient age	55.6 ± 8.2	52.6 ± 10.5	.43	48.6 ± 13.5	61.4 ± 8.0	.393	53.5 ± 9.8	53.9 ± 10.5	.916
Donor age	31.7 ± 11.6	32.8 ± 13.0	.888	44.1 ± 7.9	41.55 ± 13.6	1	35.4 ± 11.8	34.1 ± 13.3	.559
ICM recipient	5 (71.4%)	14 (50%)	.941	1 (33.3%)	4 (80%)	.293	6 (60%)	18 (54.5%)	.668
LVAD	3 (42.9%)	7 (25%)	.31	2 (66.7%)	2 (40%)	1	5 (50%)	9 (27.3%)	.252
Recipient CMV (+)	2 (28.6%)	11 (39.3%)	.523	0 (0.0%)	2 (40%)	.449	2 (20%)	13 (39.4%)	.291
Donor CMV (+)	3 (42.9%)	14 (50%)	.822	2 (66.7%)	4 (80%)	1	5 (50%)	18 (54.5%)	.904
Ischemic times (Min)	188.75 ± 65.13	183.9 ± 49.2	.706	138.0 ± 36.6	156.0 ± 7	.786	167 ± 57.5	178.7 ± 45.6	.495
Daclizumab induction	7 (100%)	24 (85.7%)	.562	3 (100%)	4 (80%)		10 (100%)	28 (84.8%)	.32
Prednisone	7 (100%)	28 (100%)	—	3 (100%)	5 (100%)		10 (100%)	33 (100%)	
Prednisone dose	8.7 ± 7.8	16.3 ± 10.2	.015	13.3 ± 2.9	13.0 ± 4.5	.845	10.1 ± 6.8	15.79 ± 9.5	.041
Maintenance regimen			.85			.4			.727
Cyclosporine	6 (85.7%)	24 (85.7%)		3 (100%)	4 (80%)		9 (90%)	28 (84.8%)	
Tacrolimus	1 (14.3%)	3 (10.7%)		0 (0.0%)	0 (0.0%)		1 (10%)	3 (9.1%)	
Sirolimus	0 (0.0%)	0 (0.0%)		0 (0.0%)	1 (20%)		0 (0.0%)	2 (6.1%)	
Cyclosporine levels (ng/ml)	437.0 ± 489.4	312.5 ± 226	.686	225.3 ± 26.6	229.25 ± 22.6	1	357.6 ± 386	300.6 ± 211	.47
Tacrolimus levels (ng/ml)	8.5 ± 0.2	11.7 ± 2.5	.083	—	—		8.5 ± 0.2	11.7 ± 2.5	.083
Sirolimus levels (ng/ml)	—	6		—	5.6		—	11.7 ± 2.5	—
Mycophenolate	6 (85.7%)	24 (85.7%)	1	3 (100%)	5 (100%)		9 (90%)	28 (84.8%)	1
Azathioprine	0 (0.0%)	0 (0.0%)	—	0 (0.0%)	0 (0.0%)	—	0 (0.0%)	0 (0.0%)	—
Simvastatin	1 (14%)	2 (7%)	.499	1 (33%)	0 (0%)	.375	2 (20%)	2 (6%)	.226
Cellular rejection (ISHLT)			.123			.315			.127
0R	4 (57.1%)	24 (85.7%)		2 (66.7%)	4 (80%)		6 (60%)	28 (84.8%)	
1R	3 (42.9%)	4 (14.3%)		0 (0.0%)	1 (20%)		3 (30%)	5 (15.1%)	
2R	0 (0.0%)	0 (0.0%)		1 (33.3%)	0 (0.0%)		1 (10%)	0 (0.0%)	
New Episodes of Rejection									
1R/1B	0 (0–1)	0 (0–4)	.3	0 (0–0)	0 (0–3)	.439	0 (0–1)	0 (0–4)	.17
2R	1 (0–1)	0 (0–2)	.59	1 (0–1)	0 (0–1)	.217	1 (0–1)	0 (0–2)	.32
2R/3A	0 (0–1)	0 (0–2)	.4	0 (0–0)	0 (0–3)	.439	0 (0–1)	0 (0–3)	.235

HTx: heart transplant; LVAD: left ventricular assist device; RT-PCR: real time polymerase chain reaction.

**Table 2:** Highest differentially expressed genes from whole genome Microarray analysis.

GS	Gene name	Gene molecular function/Gene biological process	FDR	FC	Up/Down
ANKRD22	Ankyrin repeat domain 22	Data not found	0	4.5	Up
BATF2	Basic leucine zipper transcription factor, ATF-like 2	DNA binding and transcription factor activity	0	2.6	Up
CD38	CD38 molecule	Hydrolase activity in hydrolyzing N-glycosyl compounds and lyase activity	0	2.1	Up
DCLRE1B	DNA cross-link repair 1B (PSO2 homolog, S. cerevisiae)	Nucleobase, nucleoside, nucleotide, and nucleic acid metabolic process	0	1.9	Up
EGR1	Early growth response 1	RNA binding, DNA binding, and transcription factor activity	0	4.9	Up
EPSTI1	Epithelial stromal interaction 1 (breast)	Data not found	0	3.2	Up
FAM26F	Family with sequence similarity 26, member F	Data not found	0	2.6	Up
FCGR1B	Fc fragment of IgG, high affinity Ib, receptor (CD64)	Receptor activity	0	2.8	Up
FLJ11783	Data not found	Data not found	0	0.4	Down
GNB4	Guanine nucleotide binding protein (G protein), beta polypeptide 4	GTPase activity and protein binding	0	2.3	Up
IFI44	Interferon-induced protein 44	Data not found	0	3.1	Up
IFI44L	Interferon-induced protein 44-like	Data not found	0	4.8	Up
IFI6	In multiple clusters	Immune system process	0	2.9	Up
IFIT1	Interferon-induced protein with tetratricopeptide repeats 1	Response to interferon-gamma and response to stimulus	0	7.1	Up
IFIT2	Interferon-induced protein with tetratricopeptide repeats 2	Response to interferon-gamma and response to stimulus	0	4.8	Up
IFIT3	Interferon-induced protein with tetratricopeptide repeats 3	Response to interferon-gamma and response to stimulus	0	7.4	Up
IFITM2	Interferon-induced transmembrane protein 2 (1-8D)	Immune system process	0	2.9	Up
IFITM3	Interferon-induced transmembrane protein 3 (1-8U)	Immune system process	0	2.6	Up
IFITM4P	Leukocyte Ig-like receptor, subfamily A (with TM domain), member 2	Data not found	2	2.0	Up
ISG15	Leukocyte Ig-like receptor, subfamily A (with TM domain), member 5	Ligase activity, apoptosis, and protein metabolic process	2	4.4	Up
LY6E	Lymphocyte antigen 6 complex, locus E	Data not found	0	2.8	Up
MMP8	Matrix metallopeptidase 8 (neutrophil collagenase)	Peptidase activity and protein metabolic process	0	5.2	Up
MX1	Influenza virus resistance 1, IFN-inducible protein p78 (mouse)	GTPase activity, protein binding, intracellular protein transport, and endocytosis	0	3.0	Up
OAS2	2′–5′-oligoadenylate synthetase 2, 69/71 kDa	Nucleotidyltransferase activity, nucleic acid binding, response to interferon-gamma, and response to stimulus	0	3.2	Up
PLAC8	Placenta-specific 8	Data not found	0	3.0	Up
PLSCR1	Phospholipid scramblase 1	Immune system process, lipid transport, lipid metabolic process, and blood coagulation	0	2.4	Up
RSAD2	Radical S-adenosyl methionine domain containing 2	Data not found	0	5.6	Up
SCARB2	Scavenger receptor class B, member 2	Macrophage activation, lipid transport, apoptosis, signal transduction, cell adhesion, lipid metabolic process	0	2.3	Up
SIGLEC1	Sialic acid binding Ig-like lectin 1, sialoadhesin	B cell mediated immunity, signal transduction, cell-cell adhesion, and response to stimulus	0	7.8	Up
CXCL10	Chemokine (C-X-C motif) ligand 10	Macrophage activation, cell-cell signaling, cell motion, signal transduction, and cellular defense response	1	3.4	Up
EIF2AK2	Eukaryotic translation initiation factor 2-alpha kinase 2	Immune system process, cell cycle, protein metabolic process, and response to stress	1	2.1	Up
LAP3	Leucine aminopeptidase 3	Protein metabolic process	1	3.0	Up
LCP1	Lymphocyte cytosolic protein 1 (L-plastin)	Structural constituent of cytoskeleton, cytoskeletal protein binding, and cellular component morphogenesis	1	1.9	Up
SECTM1	Secreted and transmembrane 1	Data not found	1	1.9	Up
SERPING1	Serpin peptidase inhibitor, clade G, member 1, (Angioedema)	Protein binding, peptidase inhibitor activity, and protein metabolic process	1	3.0	Up
STXBP2	Syntaxin binding protein 2	Neurotransmitter secretion, intracellular protein transport, exocytosis, and synaptic transmission	1	2.1	Up
SULT1A1	Sulfotransferase family, cytosolic, 1A, phenol-preferring, member 1	Transferase activity, sulfur metabolic process, and lipid metabolic process	1	2.1	Up
TBRG1	Transforming growth factor beta regulator 1	Receptor binding	1	0.4	Down
BLOC1S1	Biogenesis of lysosome-related organelles complex-1, subunit 1	DNA binding and transcription factor activity	2	1.9	Up
BST2	Bone marrow stromal cell antigen 2	Data not found	2	2.0	Up
CCDC84	Coiled-coil domain containing 84	Data not found	2	0.4	Down
CDC14A	CDC14 cell division cycle 14 homolog A (S. cerevisiae)	Mitosis, phosphate metabolic process, and protein metabolic process	2	0.5	Down
FLJ32679	In multiple clusters	Data not found	2	0.5	Down
FLJ38379	Hypothetical protein FLJ38379	Data not found	2	0.4	Down
HERC5	Hect domain and RLD 5	Protein metabolic process, ectoderm development, mesoderm development, and skeletal system development	2	3.6	Up

GS: Gene Symbol; FDR: false discovery rate; FC: fold change; Up/Down: regulation in advanced versus normal or mild CAV.

**Table 3:** Gene ontology categories enriched by genes expressed differentially between patients with and without advanced CAV.

Regulation in Adv-CAV	Number of genes	GO category type	Exemplary GO categories
Up-/Down-regulated	291	Biological process	GO:0006955 immune response
			GO:0002376 immune system process
			GO:0051707 response to other organism
			GO:0009607 response to biotic stimulus
			GO:0006954 inflammatory response
			GO:0050896 response to stimulus
			GO:0009615 response to virus
			GO:0009611 response to wounding
			GO:0006952 defense response
			GO:0045087 innate immune response
			GO:0009605 response to external stimulus
			GO:0042116 macrophage activation
			GO:0006950 response to stress
		Molecular function	GO:0045408 regulation of interleukin-6 biosynthetic process
			GO:0007249 I-kappaB kinase NF-kappaB cascade
			GO:0007243 protein kinase cascade
			GO:0007009 plasma membrane organization and biogenesis
			GO:0032635 interleukin-6 production
			GO:0042226 interleukin-6 biosynthetic process
			GO:0045410 positive regulation of interleukin-6 biosynthetic process
			GO:0009057 macromolecule catabolic process
			GO:0009595 detection of biotic stimulus
			GO:0007250 activation of NF-kappaB-inducing kinase
Up-Regulated	170	Biological process	None
		Molecular function	None
Down-Regulated	123	Biological process	GO:0006955 immune response
			GO:0002376 immune system process
			GO:0051707 response to other organism
			GO:0009607 response to biotic stimulus
			GO:0006954 inflammatory response
			GO:0050896 response to stimulus
			GO:0009615 response to virus
			GO:0009611 response to wounding
			GO:0006952 defense response
			GO:0045087 innate immune response
			GO:0009605 response to external stimulus
			GO:0042116 macrophage activation
			GO:0006950 response to stress
			GO:0048518 positive regulation of biological process
			GO:0002250 adaptive immune response
		Molecular function	GO:0045408 regulation of interleukin-6 biosynthetic process
			GO:0007249 I-kappaB kinase NF-kappaB cascade
			GO:0007243 protein kinase cascade
			GO:0007009 plasma membrane organization and biogenesis
			GO:0032635 interleukin-6 production
			GO:0042226 interleukin-6 biosynthetic process
			GO:0045410 positive regulation of interleukin-6 biosynthetic process
			GO:0009057 macromolecule catabolic process
			GO:0009595 detection of biotic stimulus
			GO:0007250 activation of NF-kappaB-inducing kinase
			GO:0045084 positive regulation of interleukin-12 biosynthetic process

Adv-CAV: advanced cardiac allograft vasculopathy.

**Table 4:** PCR corroboration of differentially expressed genes and Microarray-based FDR.

Gene	Up/Down	FC	*P*-value (PCR)	FDR (Microarray)
FPRL1	up	2.3	.005	3
S100A9	up	2.9	.005	3.06
CXCL10b	up	3.4	.009	1.41
CXCL10a	up	3.4	.019	1.41
PRO1073	down	0.5	.045	2.35
MMP9	up	8.0	.045	3.13
ENO1	up	1.7	.083	4.60
FAS	up	1.8	.104	4.17
